# False positivity of Rose Bengal test in patients with COVID-19: case series, uncontrolled longitudinal study

**DOI:** 10.1590/1516-3180.2020.0484.03092020

**Published:** 2020-11-13

**Authors:** Emin Gemcioglu, Abdulsamet Erden, Berkan Karabuga, Mehmet Davutoglu, Ihsan Ates, Orhan Kücüksahin, Rahmet Güner

**Affiliations:** I MD. Physician, Department of Internal Medicine, Ankara City Hospital, Ankara, Turkey.; II MD. Physician, Division of Rheumatology, Department of Internal Medicine, Ankara City Hospital, Ankara, Turkey.; III MD. Resident, Department of Internal Medicine, Ankara City Hospital, Ankara, Turkey.; IV MD. Resident, Department of Internal Medicine, Ankara City Hospital, Ankara, Turkey.; V MD. Associate Professor, Department of Internal Medicine, Ankara City Hospital, Ankara, Turkey.; VI MD. Associate Professor, Division of Rheumatology, Department of Internal Medicine, Ankara City Hospital, Ankara, Turkey.; VII MD. Professor, Department of Infectious Diseases and Clinical Microbiology, Ankara City Hospital, Ankara, Turkey.

Dear Editor

At the end of 2019, a novel coronavirus was identified as the cause of a cluster of pneumonia cases in Wuhan, China, and it spread quickly to other countries. This has led to a pandemic that has spread throughout most countries of the world in 2020. A wide variety of symptoms and signs can be seen in coronavirus disease 2019 (COVID-19) infection, such as fever, coughing, shortness of breath, arthralgia, muscle pain and diarrhea.

COVID-19 infection may show clinical or laboratory features that are similar to those of a variety of diseases. For example, it is difficult to distinguish dengue and COVID-19 because they have shared clinical and laboratory features.^1^^,^^2^ In a case report from Singapore, two patients with false-positive results from rapid serological testing for dengue, who were later confirmed to have severe acute respiratory syndrome COVID-19 infection, were reported.^3^

Brucellosis is the most common zoonosis worldwide and is a significant public health problem in many developing countries such as our country, Turkey.^4^ Brucellosis typically presents with fever, malaise and arthralgia.^5^ Common symptoms of COVID-19, such as fever, myalgia or arthralgia, can also be seen in brucellosis. The laboratory findings of these infections may be similar. Thrombocytopenia and leukopenia are common in COVID-19 and can also be seen in brucellosis. When there is a suspicion of brucellosis, the Rose Bengal test is recommended as the first test. This is a plaque agglutination test with high sensitivity that is easy to apply, has low cost and provides qualitative results. Therefore, it is frequently used as a screening test in human brucellosis cases. For this reason, we performed the Rose Bengal test on patients with COVID-19 presenting fever and arthralgia, because our country is within the endemic region for brucellosis.

In our tertiary-level medical facility, the patients received their diagnoses either through a positive polymerase chain reaction (PCR) for SARS-CoV-2 or through fulfilling three clinical criteria, including having fever and/or respiratory symptoms, compatible chest imaging findings and decreased lymphocyte count.^6^ We questioned the patients in our case series to ascertain any history of brucellosis, but found that none of them had any history relating to brucellosis or other zoonotic diseases. In eight of these patients, Rose Bengal tests were positive. These results are provided in Table 1. The median age of these eight patients was 58.5 years, and five of them were female. Arthralgia and fatigue were present in all eight patients; fever and cough were present in seven (87.5%). No patient had anosmia, ageusia, abdominal pain or diarrhea. Fourteen days after treatment for COVID-19, we performed the Rose Bengal test on all patients again, and its positivity became negative.

**Table 1:** Demographic and clinical characteristics and laboratory findings among eight patients with COVID-19

Characteristic or finding	Value in patients (n = 8)
Age – median (IQR) [years]	58.5 (47)
Female sex – n (%)	5 (62.5)
COVID-19 – n (%)	8 (100)
Duration of hospitalization – median (IQR) [days]	8.5 (5.25)
Fatigue – n (%)	8 (100)
Arthralgia - n (%)	8 (100)
Fever – n (%)	7 (87.5)
Cough – n (%)	7 (87.5)
Myalgia – n (%)	4 (50)
Dyspnea – n (%)	3 (37.5)
Headache – n (%)	2 (25)
Back pain – n (%)	1 (12.5)
Total lymphocytes – median (IQR) [per mm^3^]	1195 (677.5)
Lactate dehydrogenase – median (IQR) [U/liter]	219.5 (90.25)
C-reactive protein – median (IQR) [mg/liter]	24.5 (89.5)
Serum ferritin – median (IQR) [μg/liter]	80 (155.75)
Fibrinogen – median (IQR) [g/liter]	3.49 (2)
D-dimer – median (IQR) [mg/liter]	0.4 (1)
Positive Rose Bengal test – n (%)	8 (100)

IQR = interquartile range.

The Rose Bengal test may sometimes give a false-positive result. Various antigens obtained from *Brucella melitensis* and *Brucella abortus* are generally used in serological tests. Among these, the most widely used antigen is smooth lipopolysaccharide (S-LPS). The Rose Bengal test detects S-LPS-specific immunoglobulin M (IgM), immunoglobulin G (IgG) and immunoglobulin A (IgA) antibodies. However, this test lacks specificity to discriminate the false-positive serological reactions caused by bacteria (especially Gram-negative bacteria) sharing S-LPS epitopes with *Brucella*.^7^^,^ ^8^

It has been shown that the Rose Bengal test might have cross-reactivity with certain bacteria, including are *Francisella tularensis*, *Afipia*, *Escherichia hermannii*, *Stenotrophomonas maltophilia*, *Yersinia enterocolitica*, *Escherichia coli*, *Salmonella urbana*, *Vibrio cholerae* and others.^9^^–^^11^ Other false-positive reactions in the Rose Bengal test may be attributable to residual antibody activity from vaccinations, or to laboratory error.^12^

Our study is noteworthy in that it was the first case series to show cross-reactivity between the Rose Bengal test and the COVID-19 PCR test. Further studies are needed to explain the relationship between COVID-19 and the Rose Bengal test.

Ethics approval for this study was obtained from the Ethics Committee of a local hospital (number: E1-20-895; date: June 10, 2020).
